# Variable importance for projection (VIP) scores for analyzing the contribution of risk factors in severe adverse events to Xiyanping injection

**DOI:** 10.1186/s13020-023-00718-8

**Published:** 2023-02-13

**Authors:** Rui Zheng, Zhao Chen, Zhiyue Guan, Chen Zhao, Herong Cui, Hongcai Shang

**Affiliations:** 1 Key Laboratory of Chinese Internal Medicine of Ministry of Education and Beijing, Dongzhimen Hospital, Beijing University of Chinese Medicine, Haiyuncang Lane 5, Dongcheng District, Beijing, 100700 China; 2 Institute of Basic Research in Clinical Medicine, China Academy of Chinese Medical Sciences, No. 16, Nanxiaojie, Dongzhimennei, Dongcheng District, Beijing, China; 3 School of Life Sciences, Beijing University of Chinese Medicine, Beijing, 102488 China

**Keywords:** Xiyanping injection, Herb-drug combination, Variable importance for projection, Risk factor, Adverse events, Safety, Ribavirin, Age, Severity, Herb-drug interaction

## Abstract

**Background:**

Age and herb-drug combination are risk factors for the severity of Xiyanping injection (XYP) associated adverse events (AEs).

**Objective:**

To analyze risk factors contributing to the severity of XYP’s AEs using a variable importance for projection (VIP) method.

**Methods:**

AEs related to the use of XYP were extracted from the China National Adverse Drug Reaction Monitoring Information System (2004–2017) and classified as general or severe. Data were analyzed with respect to age and 12 herb-drug combinations, including ribavirin (RB), ceftriaxone, penicillin sodium, ambroxol hydrochloride (AH), clindamycin, cefoxitin sodium, azithromycin (AZM), ceftazidime, amoxicillin sodium/potassium clavulanate, levofloxacin hydrochloride, sodium cefazolin pentahydrate, and acyclovir according to VIP scores and correlation coefficient (Coeff).

**Results:**

A total of 21,714 AEs (general 20,660; severe 1054) related to XYP combinations were included. Using XYP alone tended to produce general AEs (All VIP = 3.124; 1.329; 1.857; 2.169; 2.400, Coeff < 0). For all set, 0–6 years old patients tend to have general AEs (VIP = 2.425, Coeff < 0), while those > 41 years old patients tend to have severe AEs (VIP = 1.180; 2.323, Coeff > 0). For 0–40 years old patients, XYP-RB combination had a greater impact on the severity of AEs (VIP = 1.158; 1.360; 1.147, Coeff > 0). For 7–17 years old patients, XYP-AZM combination tended to produce general AEs (VIP = 1.502, Coeff < 0). In individuals > 65 years old, XYP-AH combination tended to result in severe AEs (VIP = 1.232, Coeff > 0).

**Conclusions:**

VIP method was expected to effectively analyze risk factors in affecting the severity of AEs and control AEs more effectively. Age is the key factor contributing to the severity of AEs, and there are different influence directions. It is recommended that clinicians pay closer attention to the metabolic characteristics of different age groups. It is safe to use XYP alone and strictly implementing standardized operations such as medication interval and flushing will avoid undesired AEs.

## Introduction

Xiyanping injection (XYP) is a commonly used traditional Chinese medicine (TCM) preparations, made from andrographolide by sulfonation modification. It possesses strong activities of antivirus and anti-bacterium, without significant resistance [1]. It is widely combined with western medicine to treat pneumonia, psoriasis vulgaris and other diseases [2, 3]. In the treatment of psoriasis vulgaris, the add-on effects of XYP in improving efficacy, relieving symptoms in a shorter time, and reducing the hospital length of stay have been proved [4]. In the treatment of COVID-19, XYP significantly reduced the time to cough, fever resolution and virus clearance in improving the recovery of mild to moderate [5].

Since 2017, heavy concerns have been raised about the safety of XYP. Based on a study about national medical insurance database, 7 commonly prescribed concomitant (including ribavirin) medications with XYP were associated with a higher risk of suspected allergic reactions [6]. In our previous studies, age and XYP-ribavirin (RB) combination was found as risk factors for the severity of XYP-related adverse events (AEs) [7, 8].

To further explore the impact degree of risk factors on the severity of AEs, variable importance for projection (VIP) score was introduced. It is based on the partial least squares regression and used to measure the explicative power of predictor variables with respect to the response variable [9]. Mathematical indicator can rank the impact of risk factors on severity of AEs. VIP score helps clinical practitioners to control the severity of AEs more effectively. The drug-herb combination should be prescribed reasonably within the safety range to improve clinical safety.

## Methods

### Inclusion and exclusion criteria

The AEs of XYP were extracted from the National Adverse Drug Reaction Monitoring Information System (NADRMIS) from January 2004 to December 2017. Based on previous results, independent variables of interest were age and combinations [7], namely ribavirin (RB), ceftriaxone (CTR), penicillin sodium (PS), ambroxol hydrochloride (AH), clindamycin (DA), cefoxitin sodium (FOX), azithromycin (AZM), ceftazidime (CAZ), amoxicillin sodium/potassium clavulanate (AMC), levofloxacin hydrochloride (LEVH), cefazolin sodium pentahydrate (CSP) and acyclovir (ACY). More than two drugs in combination suggest a more complex relationship and was not examined in this study. Cases without available data on key factors were excluded. Personal information was not involved in our data set.

### Definition of the severity of AEs

We defined the severity of AEs and classified each AE based on the NADRMIS measures. Severe AEs included any unexpected medical occurrence included death, required hospital admission or prolongation of an existing hospital stay, or that resulted in persistent or significant disability/incapacity, cancer, congenital anomalies or birth defects, or was life-threatening. All other AEs were considered general issues [10].

### Data coding

According to the Chinese modern age classification standard, cases were stratified as children (0–6 years old), adolescents (7–17 years old), young adults (18–40 years old), middle-aged adults (41–65 years old), older adults (> 65 years old).

### Statistical analysis

VIP was used to calculate the degree of contribution of each input information to the prediction model to judge the importance of a single independent variable (risk factor) in explaining the dependent variable (the severity of AEs). The input variables of the model were age, XYP alone and the 12 XYP-combination groups. The output was the severity of AEs. Equation (1) is shown below:

1 $$VIP_{{\text{j}}} = \sqrt {\frac{k}{{\sum\limits_{h = 1}^{m} {r^{2} \left( {y,c_{h} } \right)} }}\sum\limits_{h = 1}^{m} {r^{2} \left( {y,c_{h} } \right)w_{{{\text{hj}}}}^{2} } }$$

where*, k* is an independent variable; c_*h*_ is the principal component of the relevant independent variables, r^2^(y, c_*h*_) is the correlation coefficient between the dependent variable and the principal component, indicating the explanatory ability of the principal component to y (the severity of AEs), and w_*hj*_ is the weight of the independent variable on the principal component.

**Fig. 1:**
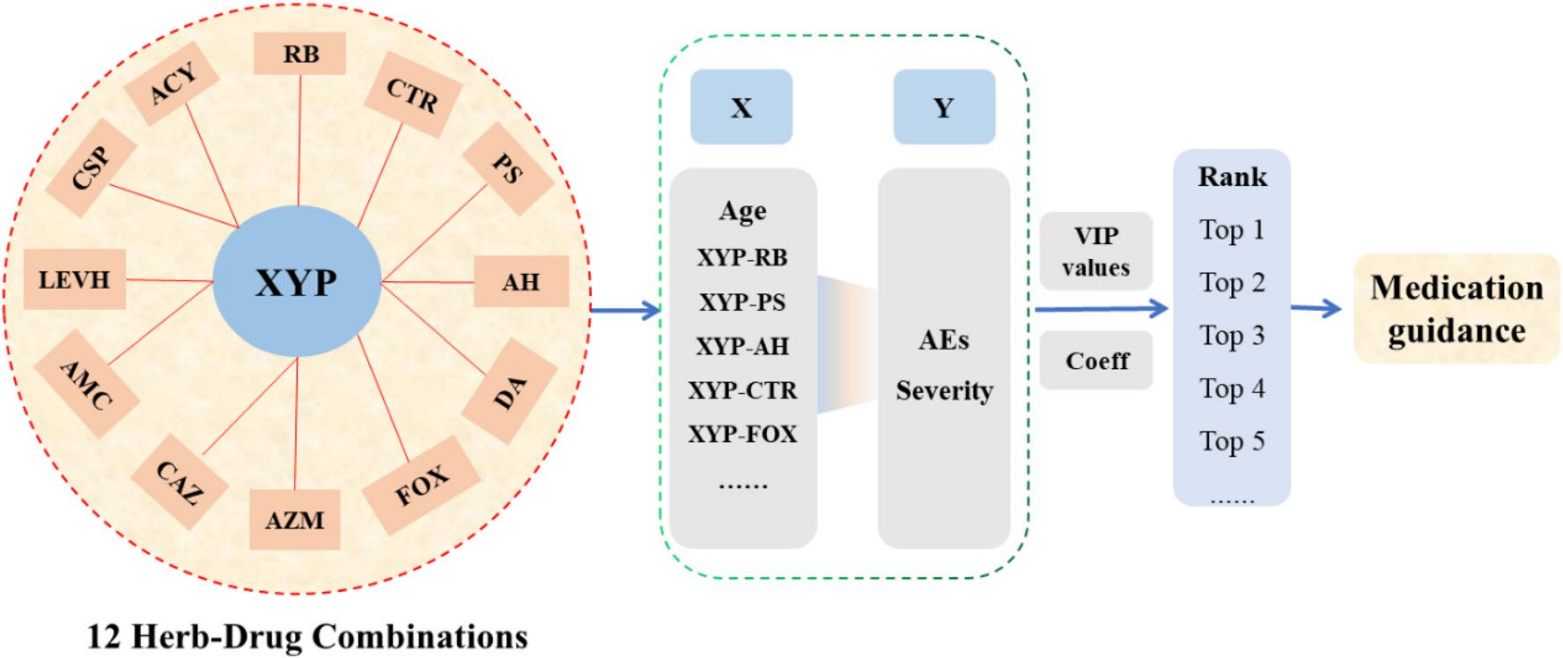
Schematic design of the variable importance for projection (VIP) method. *AH* ambroxol hydrochloride; *AMC* sodium amoxicillin and clavulanate potassium; *AZM* azithromycin; *ACY* acyclovir; *CAZ* ceftazidime; *CSP* sodium pentahydrate; *CTR* cefatriaxone; *DA* clindamycin; *FOX* cefoxitin sodium; *LEVH* levofloxacin hydrochloride; *PS* penicillin sodium; *RB* ribavirin

VIP score which is greater than 1 is the typical rule for selecting relevant variables [11] and can be considered an important contribution to Y (the severity of AEs) in the given model. If the explanatory effects of the respective variables (age, herb-drug combination) on Y (the severity of AEs) are the same, the VIP values of all independent variables are 1. The correlation coefficient (Coeff) indicates the direction of the VIP value contribution. If the Coeff > 0, the factor tends to increase the severity of AEs. If the Coeff < 0, the factor tends to reduce severity. If the VIP value is 0 or the drug is not used, there is no effects. Details are shown in Fig. 1.

## Results

### Identified studies and characteristics

We identified a total of 26,401 cases describing AEs related to the use of XYP injection in the NADRMIS. Of these, 4,687 cases did not provide any AEs indices, or other information, and were therefore excluded, resulting in 21,714 cases with eligible data. There were 20,660 general AEs and 1054 severe AEs. A full list of the distribution of AEs in different age groups is provided in Table 1.

**Table 1:** Distribution of AEs across different age groups

	Children (0–6 years old)		Adolescents (7–17 years old)		Young adults (18–40 years old)		Middle-aged adults (41–65 years old)		Older adults (> 65 years old)	
	General AEs	Severe AEs	General AEs	Severe AEs	General AEs	Severe AEs	General AEs	Severe AEs	General AEs	Severe AEs
XYP	9763(0.96)	410(0.04)	2495(0.95)	126(0.04)	2706(0.95)	146(0.05)	3062(0.95)	177(0.05)	1627(0.92)	132(0.08)
RB	92(0.93)	7(0.07)	8(0.89)	1(0.11)	10(0.83)	2(0.17)	8(0.89)	1(0.11)	7(0.88)	1(0.13)
CTR	136(0.96)	5(0.04)	33(0.94)	2(0.06)	4(0.80)	1(0.20)	7(0.78)	2(0.22)	4(1)	0(0)
PS	9(1)	0(0)	3(1)	0(0)	4(0.80)	1(0.20)	1(1)	0(0)	2(0.67)	1(0.33)
AH	23(0.96)	1(0.04)	0(0)	0(0)	0(0)	0(0)	0(0)	0(0)	6(0.86)	1(0.14)
DA	39(0.93)	3(0.07)	19(0.95)	1(0.05)	24(0.92)	2(0.08)	15(0.88)	2(0.12)	15(0.94)	1(0.06)
FOX	10(1)	0(0)	6(1)	0(0)	0(0)	0(0)	0(0)	0(0)	0(0)	0(0)
AZM	141(0.96)	6(0.04)	51(0.98)	1(0.02)	25(0.96)	1(0.04)	15(0.88)	2(0.12)	10(0.91)	1(0.09)
CAZ	57(0.93)	4(0.07)	14(0.93)	1(0.07)	1(1)	0(0)	6(0.75)	2(0.25)	8(1)	0(0)
AMC	39(0.95)	2(0.05)	5(1)	0(0)	0(0)	0(0)	3(0.75)	1(0.25)	2(1)	0(0)
LEVH	3(1)	0(0)	0(0)	0(0)	54(0.95)	3(0.05)	45(0.94)	3(0.06)	24(0.96)	1(0.04)
CSP	8(1)	0(0)	0(0)	0(0)	0(0)	0(0)	1(1)	0(0)	0(0)	0(0)
ACY	5(1)	0(0)	0(0)	0(0)	2(1)	0(0)	2(1)	0(0)	1(1)	0(0)

n = The percentage was calculated by dividing the total number of cases by the number of AE cases for each level

### VIP analysis of risk factors on age and herb-drug combination

**Fig. 2:**
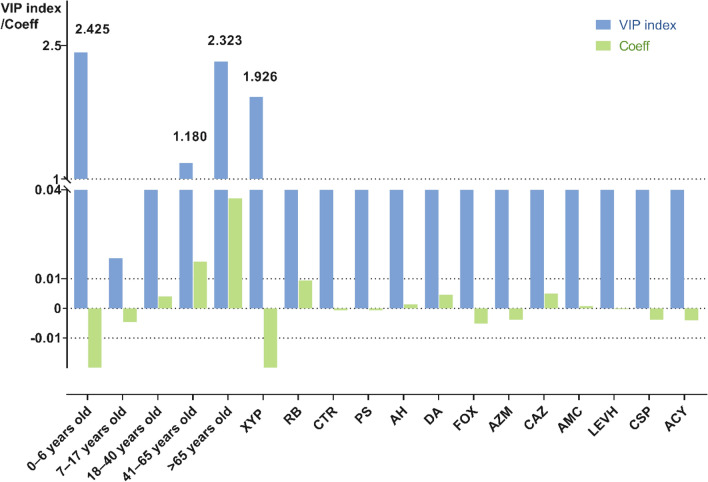
VIP values and correlation coefficients of severity of AEs (including age and herb-drug combinations)

Age, XYP alone and 12 combination drug treatment groups were included in the VIP analysis. The detailed results are shown in Fig. 2. The VIP values for the children (0–6 years old), middle-aged adults (41–65 years old), older adults (≥ 65 years old) group and XYP alone were 2.425, 1.180, 2.323 and 1.926(> 1) respectively. The correlation coefficient of the children (0–6 years old) group or XYP alone were < 0. Under the influence of 0–6 years old or XYP alone, the severity of AEs tends to be general. The correlation coefficient of the age( > 41 years old) group was > 0. AEs in patients over 41 years old tend to be severe.

### VIP analysis of herb-drug combination in 0–6 years old group

In children group (0–6 year old), the VIP score of XYP alone was 3.124 and the correlation coefficient was less than 0. This score indicates a negative impact on the severity of AEs and patients tended to have general AEs. The VIP score of XYP-RB combination was 1.158 and the correlation coefficient was more than 0, which indicated a positive impact on the severity of AEs and a tendency for more severe AEs. Detailed information is shown in Table 2.

**Table 2:** VIP value and correlation coefficient of the severity of AEs (in different age stage)

		XYP	RB	CTR	PS	AH	DA	FOX	AZM	CAZ	AMC	LEVH	CSP	ACY
0–6 years old	VIP index	3.124	1.158	0.194	0.274	0.170	0.788	0.278	0.369	0.818	0.355	0.185	0.245	0.194
	Coeff	− 0.043	0.010	− 0.005	− 0.006	− 0.001	0.007	− 0.006	− 0.002	0.006	0.001	− 0.003	− 0.005	− 0.004
7–17 years old	VIP index	1.329	1.360	0.915	0.550	–	0.559	0.778	1.502	0.751	0.711	–	–	–
	Coeff	− 0.025	0.012	− 0.002	− 0.009	–	− 0.004	− 0.012	− 0.025	0.002	− 0.011	–	–	–
18–40 years old	VIP index	1.857	1.147	0.939	0.939	–	0.570	–	0.044	–	–	0.380	–	–
	Coeff	− 0.041	0.025	0.021	0.021	–	0.007	–	− 0.006	–	–	− 0.002	–	–
41–65 years old	VIP index	2.169	0.414	0.914	–	–	0.611	–	0.591	0.979	0.650	0.431	–	–
	Coeff	− 0.067	0.008	0.026	–	–	0.012	–	0.012	0.029	0.020	0.000	–	–
> 65 years old	VIP index	2.400	0.606	0.169	–	1.232	0.267	–	0.441	0.240	–	0.050	–	–
	Coeff	− 0.092	0.009	− 0.015	–	0.037	− 0.018	–	0.001	− 0.021	–	− 0.020	–	–

The green flag indicates that the correlation coefficient is < 0, and that this factor had a negative impact on the severity of the AE and tended to result in general AEs. The red flag indicates that the correlation coefficient > 0, and this factor had an active impact on the severity of the AE and tended to result in a severe AE. The VIP value is 0 or the drug is not used, which is not shown

### VIP analysis of herb-drug combinations in the 7–17 years old group

In 7–17 years old group, the VIP values of XYP alone and XYP-AZM combination were 1.329 and 1.502 respectively, and the correlation coefficient was less than 0, which indicated a negative impact on the severity of AEs and a tendency for general AEs. The influence of the XYP-AZM combination was greater than that of the XYP alone. The VIP score for XYP-RB combination was 1.360 and the correlation coefficient was > 0. They had a promoting effect on the severity of AEs and tended to develop severe AEs.

### VIP analysis of herb-drug combinations in the 18–40 years old group

In the 18–40 years old group, the VIP score for XYP alone was 1.857 and the correlation coefficient of XYP alone was < 0, indicating a negative effect on the severity of AEs and a tendency for have general AEs. VIP value of XYP-RB combination was 1.147 and the correlation coefficient was > 0. Under the influence of XYP-RB combination, there was a higher tendency to for more severe AEs.

### VIP analysis of herb-drug combinations in the 41–65 years old group

The VIP score for XYP alone was 2.169 and the correlation coefficient was < 0, indicating that this factor had a negative impact on the severity of the AEs and tended to exhibit general AEs.

### VIP analysis of herb-drug combination in the > 65 years old group

The contribution of combination drugs to the severity of AEs was analyzed in the > 65 years old group. The VIP score for XYP alone was 2.400 and the correlation coefficient was < 0, indicating that this factor had a negative impact on the severity of AEs and tended to have general AEs. The VIP score for XYP-AH combination was 1.232 and the correlation coefficient was > 0. Under the influence of these combination, there was a tendency for more severe AEs.

## Discussion

Based on the VIP scores and the correlation coefficients, the results showed that the using XYP alone tended to produce general AEs. Age is the key factor contributing to the severity of AEs, and there are different influence directions. 0–6 years old patients tend to have general AEs, while those > 41 years old patients tend to have severe AEs. For 0–40 years old patients, XYP-RB combination had a greater impact on the severity of AEs. For 7–17 years old patients, XYP-AZM combination tended to produce general AEs. In individuals > 65 years old, XYP-AH combination tended to result in severe AEs.

### Introducing the VIP approach into clinical medical statistics

In practical applications, VIP scores are useful in understanding the predictor variables of the X space that best explain the variance of Y [12]. It often applied to the analysis of large data sets in analytical chemistry [13], medical statistics [14], pharmaceutical engineering [15, 16]. VIP analysis method used in the analysis of clinical risk factors, which helps to understand the influence of risk factors on the severity of AEs. Applying this method in this study, we found that XYP alone had better security. XYP-RB combination plays a greater role in the severity of AEs. Therefore, we highly recommend using a variable selection method such as VIP to assess risk factors that affect AE indicators to more targeted improve clinical safety.

### Age is a key factor in the safety of the combination treatments

Age is the key factor affecting the occurrence of adverse reactions [17, 18][19]. Based on clinical safety surveillance of 296,200 cases, age was also a related risk factor for TCM injections [20]. Patients at different ages exhibit different sensitivities to different combinations of drugs. According to the annual report of national adverse drug reaction monitoring (2021), the age of 31.2% of patients with AEs was over 65 years old in China [21].

Age has an influence on physiological systems, for instance, different stages of immune cells, such as antigen-naïve CD8^+^ T cells exhibit marked proliferative dysfunction in advanced age [22]. In this study, 0–6 years old patients tend to have general AEs, while those > 41 years old patients tend to have severe AEs. XYP-AZM/AH combination has different effects at different stages. Age may regulate unknown drug metabolism. It is necessary to perform a targeted clinical safety analysis of different drugs according to the metabolic and immune characteristics of age groups.

### AEs in children

Children, especially infants, are not fully developed. They are relatively sensitive to drugs and are more likely to develop AEs [23]. XYP is widely used in pediatric diseases, including pediatric upper respiratory tract infections [24] and viral pneumonia [25].A retrospective study analyzed data from center for adverse drug reaction monitoring of Fujian, the AEs of XYP were mainly concentrated in patients under 14 years of age, especially infants under 4 years of age, which represented 67.1% [26].

The XYP-western medicine combination therapy achieved a better therapeutic efficacy in treating severe hand, foot and mouth disease than the western medicine therapy alone [27]. However, XYP pose graver risks to the children than the adult [28]. How to achieve the best therapeutic effect under safe conditions is the key to the problem. Children should undergo targeted pharmacokinetic tests of specific drugs and dosages should not simply be the converted dose from the adult.

### AEs of the XYP-RB combination tended to be more severe

Before the drug leaves the factories, injection safety tests are necessary, including histamine substances and allergic reactions. Type I allergic reactions probably be excluded. Based on clinical features, AEs of XYP-RB combination may be a pseudo-allergic reaction [8]. Pseudo-allergic reaction is a common adverse reaction of TCM injection. The mechanism is mainly characterized by a change in vascular permeability [29].

For patients in 0–40 years old, XYP-RB combination was a major factor associated with increasing severity of AEs. Older adults have more complex effects of drug combination, because metabolism is slowed down and due to the concomitant diseases associated with ageing. In patients older than 65 years, the contribution of the XYP-RB combination tended to lighten the severity of AEs. New interactions of drug targets or metabolism, or other unknown factors may contribute to reduce AEs.

For instance, Fasudil is a drug commonly used to improve cerebral microcirculation and promote nerve regeneration, but it is also a ROCK inhibitor [30]. Blocking RhoA/ROCK signaling pathway can reduce vascular permeability and inhibit the occurrence of a pseudo-allergic reaction [31]. Individuals over 65 years old are prone to cerebrovascular diseases. It’s possible that similar drugs were used and inhibited the pseudo-allergic reaction.

Cytochrome P450 is the main enzyme system of drug metabolism and one of the key targets of drug AEs. XYP regulates metabolism by inhibiting P450 enzymes. For example, XYP significantly increases all pharmacokinetic parameters of glibenclamide by inhibiting CYP3A4 [32]. Studies have found that Reduning injection, a TCM injection, inhibits the gene expression of CYP1A2, CYP2A6, and CYP2B6. Changing the pharmacokinetics of RB, thus greatly increases the plasma concentration of RB in the liver and initiates side effects [33]. XYP is similar to Reduning injection on the TCM efficacy. Whether the XYP-RB combination has similar effects and why it exerts different effects in different age groups needs to be further explored.

### Corresponding countermeasures

#### (1) Standardization of the route of administration

XYP alone tends to have general AEs, suggesting that XYP alone may have a good safety. XYP-RB combination is more effective in the treatment of severe hand, foot, and mouth diseases [34]. Therefore, how to balance and improve the curative effect should focus on ensuring safety. *The Basic Principles for the Clinical Use of Traditional Chinese Medicine Injections* specify that TCM injections should be used as monotherapy. If they really need to be used in combination with other drugs, the interval between two doses should be carefully considered. It is recommended to flush the infusion tube during dressing changes. It is emphasized that standardizing drug use according to the instructions is a feasible way to ensure drug safety.

#### (2) Strengthening mechanism research

Combining TCM and Western medicine is a common clinical approach, but how to reasonably evaluate clinical safety has always been a dilemma. Finding key influencing factors helps to effectively avoid AEs. VIP analysis showed that the XYP-RB combination had a significant contribution to the severity of AEs. We refer to the safety evaluation method ‘Feature target correlation method’ to extract the clinical characteristics of AEs and correlate the target mechanisms. This will help to avoid same types of combinations and will improve drug safety [35].

## Limitations

There are several limitations to this study. First, as a continuation of previous researches, we were only access AE information related to XYP in the NADRMIS database from 2004 to 2017 and only 12 commonly used clinical combinations were analyzed. This study only introduces VIP method and explains the clinical significance. The findings do not reflect the entire history of XYP use. Second, AE reports may be influenced by factors not included in the NADRMIS, such as genetic characteristics that cannot be addressed in our analyses. Third, this study only analyzes the phenomenon, and the analytical ability of this method to address additional factors and to understand any underlying mechanisms are required further verification. Further studies should evaluate why severe AEs are attributed to treatment combination rather than to a single drug/herb.

## Conclusions

VIP scores are useful in evaluating risk factors that affect outcome indicators in clinical studies. It helps to rank the risk factors and control the severity of AEs more effectively. Age is a key factor in the severity of AEs to XYP. For aged 0–40 years old patients, XYP-RB combination tends to worsen AEs. It is recommended that clinicians pay closer attention to the metabolic characteristics of different age groups. XYP alone tended to produce general AEs. It is safe to use XYP alone and strictly implementing standardized operations such as medication interval and flushing will avoid undesired AEs.

## Data Availability

All data used in the presented study can get from the corresponding author upon request.

## Abbreviations

AEs: Adverse events
AH: Ambroxol hydrochloride
AMC: Amoxicillin sodium and clavulanate potassium
AZM: Azithromycin
CAZ: Ceftazidime
ACY: Acyclovir
CSP: Cefazolin sodium pentahydrate
Coeff: Correlation coefficient
CTR: Ceftriaxone
DA: Clindamycin
FOX: Cefoxitin sodium
LEVH: Levofloxacin hydrochloride
NADRMIS: National Adverse Drug Reaction Monitoring Information System
PS: Penicillin sodium
RB: Ribavirin
TCM: Traditional Chinese medicine
VIP: Variable importance for projection
XYP: Xiyanping injection
